# Prognostic role of IL-34 in sepsis and sepsis-induced acute lung injury: preliminary results and future directions

**DOI:** 10.3389/abp.2025.13958

**Published:** 2025-03-19

**Authors:** Run Cai, Jianke Ren, Chenwei Zhou, Yuxin Liu, Jianlei Tang, Weiyan Cui, Yongmin Yan, Sheliang Xue, Yanjuan Zhou

**Affiliations:** ^1^ Department of Respiratory and Critical Care Medicine, Wujin Hospital Affiliated with Jiangsu University, Changzhou, China; ^2^ National Health Commission Key Lab of Reproduction Regulation, Shanghai Engineering Research Center of Reproductive Health Drug and Devices, Shanghai Institute for Biomedical and Pharmaceutical Technologies, Shanghai, China; ^3^ Department of Respiratory and Critical Care, Changzhou No. 7 People’s Hospital, Changzhou, China; ^4^ Intensive Care Unit, Wujin Hospital Affiliated with Jiangsu University, Changzhou, China; ^5^ Central Laboratory, Wujin Hospital Affiliated with Jiangsu University, Changzhou, China; ^6^ Department of Cardiology, Wujin Hospital Affiliated with Jiangsu University, Changzhou, China

**Keywords:** IL-34, sepsis, ALI, prognosis, risk stratification

## Abstract

**Objective:**

This study aimed to evaluate the potential of interleukin-34 (IL-34) as a novel biomarker for predicting mortality in sepsis patients, with a specific focus on those with sepsis-induced acute lung injury (ALI).

**Methods:**

This prospective cohort study enrolled 115 sepsis patients admitted to the intensive care unit (ICU). The patients were divided into survival and non-survival groups, as well as ALI and non-ALI subgroups. Serum levels of IL-34, in conjunction with other established biomarkers such as interleukin-6 (IL-6), C-reactive protein (CRP), and lactate, were measured and analyzed. Statistical analyses, including receiver operating characteristic (ROC) curves, Kaplan-Meier survival curves and Cox regression models, were used to determine the prognostic significance of IL-34.

**Results:**

Serum IL-34 levels were significantly elevated in sepsis patients compared to healthy controls, and they were also higher in non-survival group compared to survival group (*p* < 0.05). Additionally, IL-34 levels exhibited a positive correlation with sepsis severity, as indicated by APACHE II and SOFA scores. Kaplan-Meier survival curves and multivariate COX regression analysis revealed that IL-34 is an independent risk factor for death within 28 days of sepsis. The serum IL-34 level in the ALI group was significantly higher than that in the non-ALI group, particularly in severe cases (*p* < 0.05). However, the prognostic value of IL-34 in sepsis-induced ALI requires further investigation.

**Conclusion:**

IL-34 shows promise as an independent prognostic factor in sepsis patients and may enhance risk stratification, especially in those with sepsis-induced ALI.

## Introduction

Sepsis represents a significant public health challenge, characterized by high morbidity and mortality rates of up to approximately 30%, and poses a serious threat to patients’ lives in both general hospital wards and intensive care units (ICU) (Wang et al., 2021). Sepsis is defined as life-threatening organ dysfunction resulting from an uncontrolled host immune response to infection (Singer et al., 2016). In essence, this dysregulated immune response not only fails to protect the body from infection, but also exacerbates the systemic inflammatory response, leading to damage across multiple organs. Among the various organs affected by multi-organ dysfunction in sepsis, the lungs are often the earliest and most susceptible to invasion (Sadowitz et al., 2011; Park et al., 2019). Research indicates that 25%–50% of sepsis patients will induce acute lung injury (ALI), with mortality rates in these patients potentially reaching as high as 40% (Sevransky et al., 2004; Rubenfeld et al., 2005). This highlights that ALI is a particularly dangerous complication during the course of sepsis and has a substantial impact on patient prognosis. Clinically, patients with ALI typically present with systemic inflammatory response syndrome, characterized by refractory hypoxemia and severe respiratory distress (Martin and Silverman, 1992; Jin et al., 2020). These symptoms not only significantly impair the patient’s quality of life but also necessitate advanced medical support, such as mechanical ventilation and intensive care interventions to sustain life. For these patients, timely risk stratification can reduce mortality.

In the field of sepsis research, various biomarkers have been extensively studied and established as important indicators for detection and prognosis. These biomarkers include interleukin 6 (IL-6), C-reactive protein (CRP), interleukin 8 (IL-8), tumor necrosis factor-α (TNF-α), and interleukin 1β (IL-1β), along with their combinations (Carcò et al., 2022; Li, 2024; Zeng et al., 2022). Our study specifically focuses on interleukin 34 (IL-34) due to its novel role in regulating immune responses, particularly in the context of sepsis. IL-34, a cytokine first described in 2008 (H. Lin et al., 2008), is secreted by the monocyte-macrophage system, epithelial cells, and fibroblasts, and is continuously expressed in various tissues and organs, including the human heart, lungs, and spleen (Muñoz-Garcia et al., 2021). Numerous previous studies have demonstrated that IL-34 plays a significant role in various cancers, autoimmune diseases, and inflammatory conditions (Preisser et al., 2014; Zhou R. et al., 2016; Zhou S. et al., 2016; Zwicker et al., 2015). Additionally, research has indicated that IL-34 is associated with respiratory diseases such as pneumonia and interstitial lung disease (Kuzumi et al., 2018; Tang et al., 2024). Recent studies have underscored the relationship between IL-34 and sepsis, illustrating its potential role in mediating immune responses. For instance, Jacobs and Wiersinga (2018) discussed IL-34 as an emerging factor in sepsis, proposing that it may influence pathogenic processes related to systemic inflammation and organ dysfunction. Furthermore, Lin et al. (2018) provided compelling evidence that IL-34 contributes to improved survival and bacterial clearance in polymicrobial sepsis, emphasizing its importance in the host response to infection. It is important to clarify that this study does not seek to diminish the significance of established biomarkers; rather, it aims to explore the novel and understudied role of IL-34 in sepsis.

Currently, there is limited direct data regarding the relationship between IL-34 and sepsis patients, and no reports have been published on the role of IL-34 in patients with sepsis-induced ALI. Therefore, this study aimed to investigate the potential of IL-34 as a biomarker for both sepsis and sepsis-induced ALI. We hypothesized that elevated levels of IL-34 would correlate with the severity of sepsis and sepsis-induced ALI, thereby serving as a reliable prognostic marker. The primary objective of this study was to determine whether IL-34 can predict outcomes in ICU patients suffering from sepsis and sepsis-induced ALI.

## Patients and methods

### Basic information

This prospective cohort study was conducted with 115 sepsis patients who were consecutively admitted to the ICU at Changzhou Wujin People’s Hospital through October 2022 to August 2024. The cohort consisted of 66 male and 49 female patients, with a mean age of 76 years (ranging from 70 to 82 years). The data collected included demographic and clinical parameters such as age, gender, body temperature, pulse rate, mean arterial pressure, underlying health conditions, white blood cell count, creatinine levels, total bilirubin levels, SOFA score, and APACHE II score. In addition, we included 28 healthy individuals from the physical examination center as controls during the same period. The study received ethical approval from the Ethics Committee of Changzhou Wujin People’s Hospital, and informed consent was obtained from all participants or their legal guardians.

### Inclusion criteria

Patients were eligible for inclusion if they had a confirmed diagnosis of sepsis according to the Sepsis 3.0 criteria (Singer et al., 2016), and ALI was diagnosed using the 1994 American-European Consensus Conference (Bernard et al., 1994). All participants were adults aged 18 years or older.

### Exclusion criteria

Exclusion criteria included patients under 18 years old, pregnant women, patients with severe immunosuppression, and patients with malignant tumors.

### Case grouping

During the study, the 115 sepsis patients were categorized into two subgroups: the survival group (n = 82) and the non-survival group (n = 33). After excluding five patients with lung injuries from other causes, the remaining 110 sepsis patients were further divided into two groups: the non-ALI group (n = 36) and the ALI group (n = 74). The ALI group was then stratified into mild-to-moderate (n = 62) and severe (n = 12) subgroups.

### Cytokine assays

Cytokine levels were assessed by collecting blood samples at 24, 72, and 120 h post-diagnosis, with sampling intervals of 1–2 days. The serum IL-34 levels were measured using enzyme-linked immunosorbent assay (ELISA), with reagents supplied by Jianglai Biological Company. The assays were conducted in strict accordance with the manufacturer’s protocols.

### Statistical analysis

The normality of the distribution for quantitative variables was assessed using the Kolmogorov-Smirnov test, with a threshold of P > 0.10 indicating a normal distribution. Data that were normally distributed were reported as mean ± standard deviation (SD), while non-normally distributed data were presented as median with interquartile range (IQR). Qualitative variables were described using frequencies and percentages. For the comparison of continuous variables between two groups, the Student’s t-test was employed for normally distributed data, whereas the Mann-Whitney U test was used for data not following a normal distribution. Categorical variables were analyzed using the Chi-square test, Fisher’s exact test, or the McNemar test, depending on the nature of the data. Correlations between IL-34 levels and other clinical or biomarker variables were explored using Spearman’s correlation coefficients. First, logistic regression was used to calculate the predicted values of the combination of IL-34 levels and SOFA scores. Subsequently, the Receiver Operating Characteristic (ROC) curve was used to evaluate the clinical prognostic value of IL-34, SOFA scores, and their combination in predicting the prognosis in sepsis patients. The optimal cutoff value for IL-34 was determined using the Youden index. Kaplan-Meier survival curves were generated to compare groups based on IL-34 levels, and differences were assessed using the log-rank test. To identify independent risk factors associated with 28-day mortality, a multivariate Cox proportional hazards regression model was utilized. All statistical analyses were performed using SPSS version 26.0 (IBM SPSS, United States), and a two-tailed P value of less than 0.05 was considered to indicate statistical significance.

## Results

### High expression of IL-34 in sepsis patients and non-survival group and dynamic changes of IL-34

**FIGURE 1:**
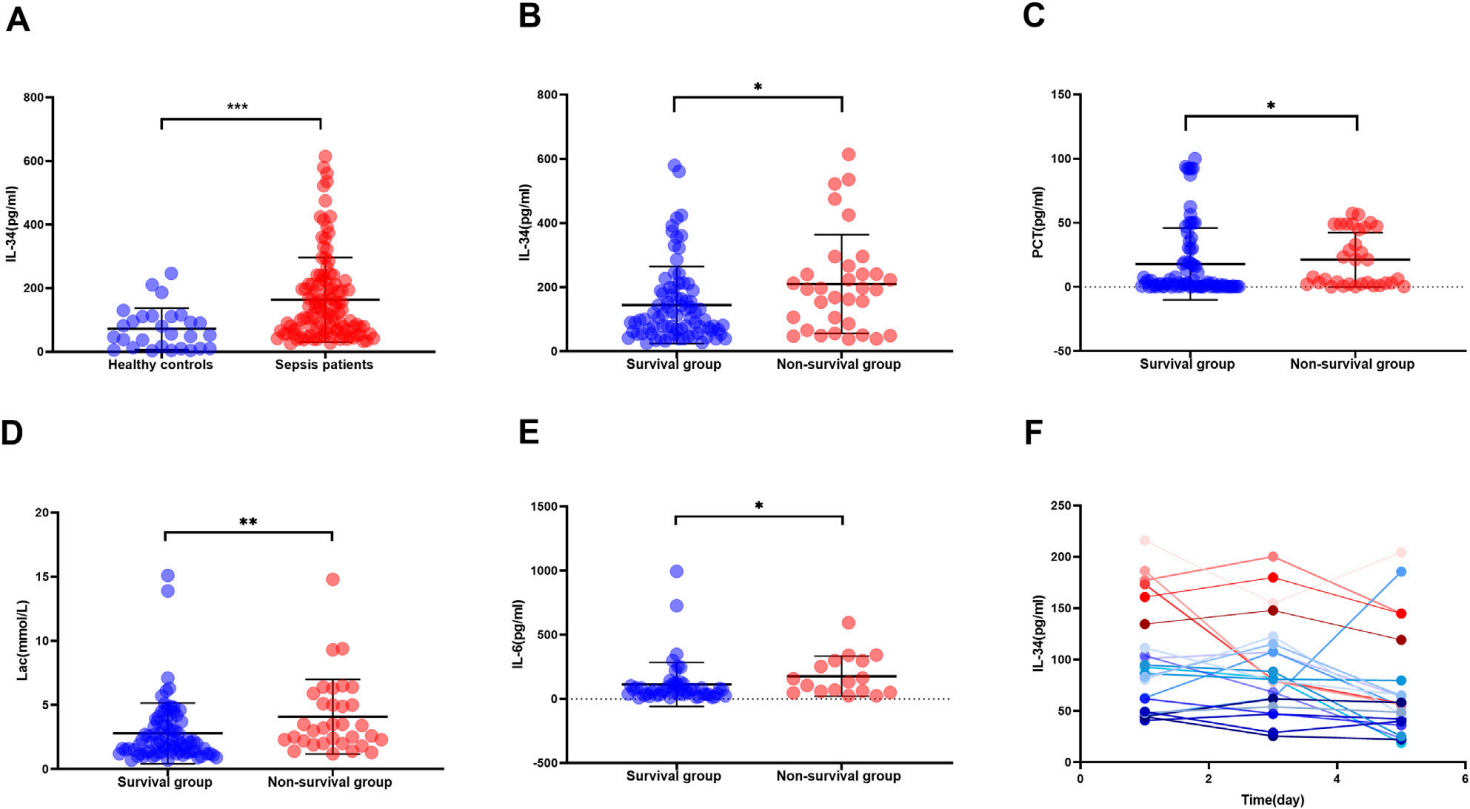
High expression of IL-34 in sepsis patients and non-survival group and dynamic changes of IL-34. **(A)** Serum IL-34 levels are significantly higher in sepsis patients (n = 115) compared to healthy controls (n = 28). **(B–E)** IL-34, PCT, Lac, and IL-6 are highly expressed in the serum of non-survival group. [survival group (n = 82) and non-survival group (n = 33)] **(F)**. Changes in serum IL-34 levels measured in 22 sepsis patients on the first, third, and fifth days of admission (*p < 0.05, **p < 0.01, ***p < 0.001).

We measured the levels of IL-34 in the serum of 115 sepsis patients and 28 healthy individuals, finding that the levels of IL-34 in sepsis patients were significantly elevated compared to those in healthy individuals (Figure 1A).

Subsequently, we divided 115 sepsis patients into a survival group [82 patients (71.3%)] and a non-survival group [33 patients (28.7%)] according to whether they died within 28 days, and recorded the baseline clinical status of these two groups of patients. Characteristics include age, gender, underlying diseases, vital signs, blood results, GCS score, APACHE II score, SOFA score, and the proportion of ALI patients. (Table 1). Our analysis revealed that the GCS score, APACHE II score, SOFA score, and the proportion of ALI patients were significantly higher in the non-survival group compared to the survival group (all *p* < 0.05). Then, we further tested and found that the serum IL-34, IL-6, PCT, and Lac levels of patients in the non-survival group were also significantly higher than those in the survival group (Figures 1B–E).

**TABLE 1:** Clinical characteristics of sepsis patients in survival and non-survival groups.

Parameter	Survival group (n = 82)	Non-survival group (n = 33)	*P* value
Age (years)	76 (69–80)	78 (71–85)	0.094
Gender (male/female)	59/23	24/9	0.908
Underlying diseases, n (%)			
Coronary heart disease	5 (6.1%)	4 (12.1%)	0.443
Hypertension	48 (58.5%)	22 (66.7%)	0.419
Type 2 diabetes	24 (29.3%)	13 (39.4%)	0.293
Chronic renal failure	3 (3.7%)	3 (9.1%)	0.471
Endotracheal intubation	44 (53.7%)	25 (75.8%)	**0.029**
Mean arterial pressure (mmHg)	87.7 (78.0–96.7)	85.7 (67.3–97.8)	0.203
Respiratory rate (per minute)	20 (17–23)	20 (18–25)	0.449
Heart rate (bpm)	96 (86–107)	101 (80–118)	0.409
Temperature (°C)	36.8 (36.5–37.4)	36.7 (36.5–37.6)	0.884
White blood count (10^9^/L)	12.5 (9.7–19.5)	16.3 (9.9–23.6)	0.281
Platelet count (10^9^/L)	174 (121–241)	198 (130–226)	0.711
Hematocrit (%)	33.6 ± 7.7	33.1 ± 8.6	0.766
Total bilirubin (μmmol/L)	18.3 (12–29.9)	17.8 (12.6–33.5)	0.514
Creatinine (μmmol/L)	97.1 (70.0–145.0)	98.5 (80.4–147.0)	0.510
CRP (mg/L)	110.3 (56.9–182.9)	128.7 (47.5–172.5)	0.961
GCS score	11 (9–15)	9 (6–15)	**0.032**
SOFA score	9 ± 3	12 ± 4	**0.001**
APACHE Ⅱ score	20 (15–25)	27 (20–33)	**0.004**
the proportion of ALI patients	61.5%	81.3%	**0.045**

The bolded *p*-value indicates a statistically significant difference (*p* < 0.05).

We recorded serum IL-34 levels in 22 sepsis patients on the first, third, and fifth days to monitor changes. Preliminary observations indicated that IL-34 levels decreased in the majority of patients (86.4%) after 5 days compared to the first day, and none of these patients died within 28 days. In contrast, levels increased in a minority of patients (13.6%) (Figure 1F). Further analysis of these observations in relation to specific patient conditions indicated that, among the three patients whose IL-34 levels increased after 5 days, two had a prognosis of death, while the third case involved a patient who was hospitalized for over 1 month. Additionally, bacterial cultures from the body fluids of these three patients were all positive, identifying *Streptococcus* pneumoniae, Gram-negative bacilli, and Gram-positive bacilli.

### Correlation of IL-34 levels with scores reflecting sepsis severity and related blood biomarkers

**FIGURE 2:**
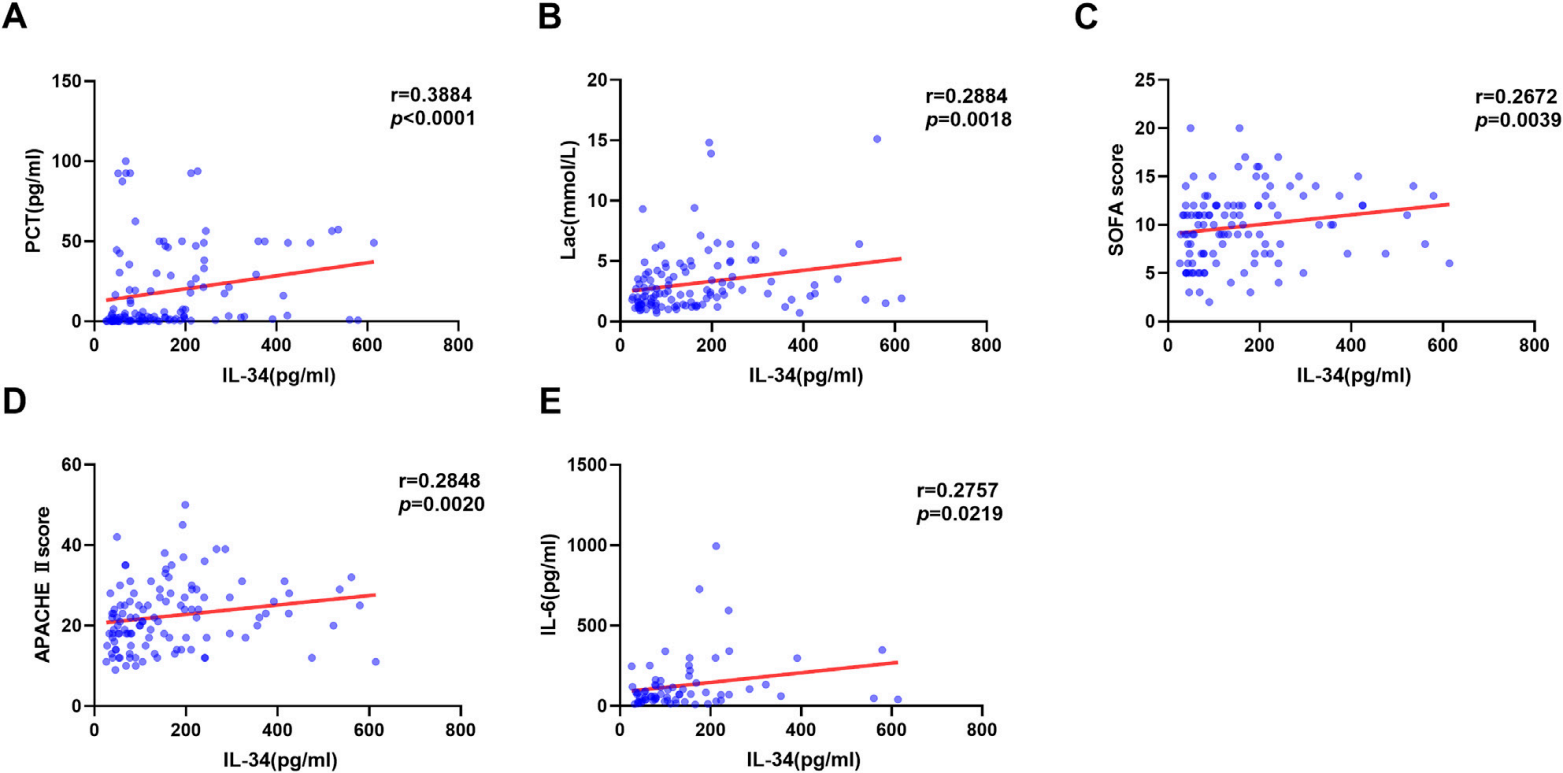
Correlation between serum IL-34 levels and PCT **(A)**, Lac **(B)**, SOFA scores **(C)**, APACHE II scores **(D)** and IL-6 levels **(E)** respectively (n = 115).

Spearman correlation analysis was employed to examine the relationship between IL-34 levels and scores indicative of sepsis severity, as well as associated inflammatory biomarkers. The findings revealed a positive correlation between IL-34 levels and PCT, Lac, SOFA scores, APACHE II scores and IL-6 levels (Figure 2).

### The prognostic value of serum IL-34 levels in sepsis patients

**FIGURE 3:**
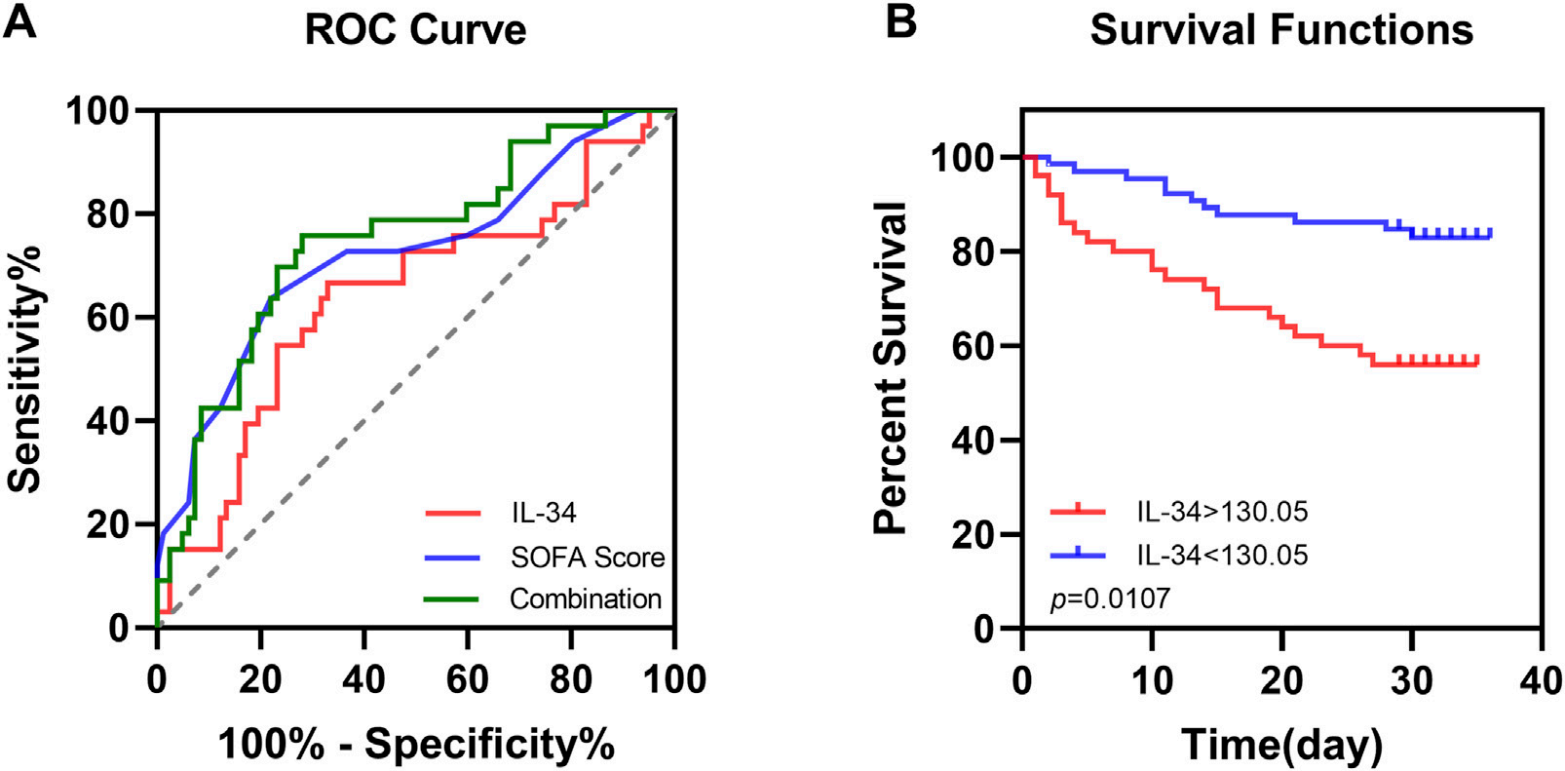
Elevated serum IL-34 levels are an independent risk factor for mortality in sepsis patients at 28 days. **(A)** ROC curve of IL-34 (red line), SOFA score (blue line) and their combination (green line) to predict sepsis patients mortality. **(B)** Survival curves for sepsis patients with IL-34 levels greater than 130.05 pg/mL (red line) and those with IL-34 levels less than 130.05 pg/mL (blue line). (p = 0.0107).

The association between IL-34 levels and 28-day mortality was assessed using both ROC curve analysis and the Kaplan-Meier method. The ROC analysis revealed that the AUC for IL-34 in predicting 28-day mortality was 0.640, with a sensitivity of 66.7% and a specificity of 67.1%. When the levels of IL-34 were combined with the SOFA score, the sensitivity increased to 0.758, and the AUC rose to 0.722. This finding indicates an improvement in clinical screening sensitivity and predictive value when compared to the SOFA score alone (Figure 3A; Table 2). Additionally, the Kaplan-Meier survival analysis demonstrated that patients with IL-34 levels below 130.05 pg/mL had a significantly higher survival rate compared to those with levels above 130.05 pg/mL (*p* = 0.0107, log-rank test; Figure 3B). We conducted a univariate Cox regression analysis using age, gender, IL-34, IL-6, PCT, CRP, LAC, GCS score, SOFA score, and APACHE II score as independent variables. The analysis revealed that IL-34, LAC, GCS score, SOFA score, and APACHE II score were significantly associated with mortality. These five variables were subsequently included in a multivariate Cox regression model. The multivariate analysis showed that both IL-34 and SOFA scores were significantly associated with mortality (Table 3). These findings further demonstrated that IL-34 levels was an independent risk factor for mortality in sepsis patients and could potentially serve as a biomarker for predicting 28-day mortality.

**TABLE 2:** IL-34, SOFA score, and their combined prognostic value in sepsis.

Parameter	Cutoff value	AUC	SE	CI(95%)	*P* value	Sensitivity	Specificity
IL-34	152.78	0.640	0.060	(0.522 ∼ 0.757)	<0.05	0.667	0.671
SOFA score	11.5	0.722	0.056	(0.612 ∼ 0.833)	<0.05	0.636	0.780
Combination of both above	0.28	0.748	0.052	(0.647∼0.849)	<0.05	0.758	0.720

**TABLE 3:** Cox proportional hazards model for mortality prediction.

Variable	Univariate cox model		Multivariate cox model one	
	HR (95%CI)	*P* value	HR (95%CI)	*P* value
Age	NA	0.098	NA	NA
Gender	NA	0.674	NA	NA
GCS score	0.905 (0.831–0.985)	**0.021**	NA	0.864
SOFA score	1.221 (1.109–1.344)	**0.000**	1.211 (1.039–1.411)	**0.014**
APACHE Ⅱ score	1.064 (1.025–1.104)	**0.001**	NA	0.463
CRP	NA	0.840	NA	NA
PCT	NA	0.481	NA	NA
LAC	1.144 (1.043–1.254)	**0.004**	NA	0.208
IL-6	NA	0.993	NA	NA
IL-34	3.200 (1.549–6.609)	**0.002**	2.2 (1.010–4.793)	**0.047**

NA, Not Applicable.

The bolded *p*-value indicates a statistically significant difference (*p* < 0.05).

### IL-34 is highly expressed in the ALI group and its risk stratification and prognostic value

**FIGURE 4:**
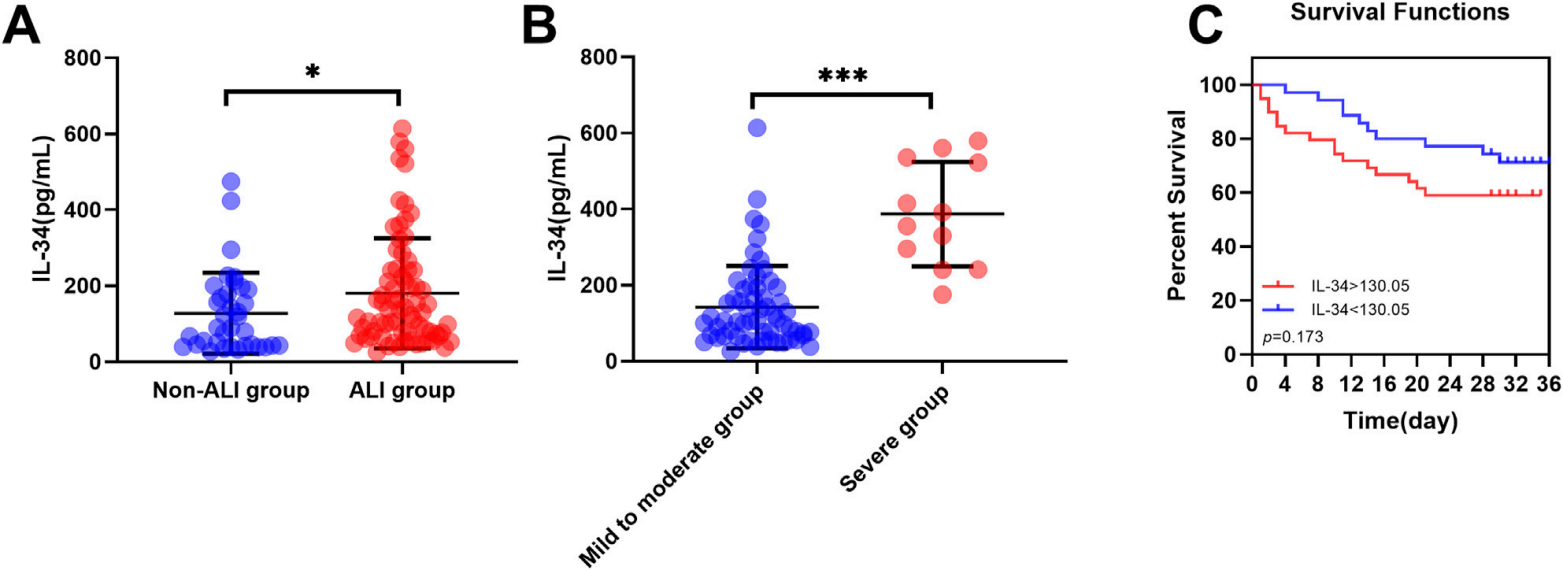
IL-34 is highly expressed in the ALI group and its risk stratification and prognostic value. **(A)** The serum IL-34 level in the ALI group (n = 74) was significantly higher than that in the non-ALI group (n = 36). **(B)** Within the ALI group, serum IL-34 levels were significantly higher in the severe subgroup (n = 62) compared to the mild-to-moderate subgroup (n = 12). **(C)** Survival curves for sepsis-induced ALI patients with IL-34 levels above 130.05 pg/mL (red line) and below 130.05 pg/mL (blue line) (p = 0.173) (*p < 0.05, ***p < 0.001).

The aforementioned study indicated that the proportion of ALI patients in the non-survival group (81.3%) was significantly higher than that in the survival group (61.5%) (Table 1). To further investigate the relationship between IL-34 and sepsis-induced ALI, we excluded five patients with lung injuries due to other causes. Following this exclusion, the remaining 110 patients were classified into two groups: the non-ALI group (n = 36) and the ALI group (n = 74). The baseline clinical characteristics of both patient groups included age, gender, underlying diseases, tracheal intubation, vital signs, oxygenation index, blood results, GCS score, APACHE II score, SOFA score and the proportion of non-survival patients (Table 4). After analysis, the proportions of endotracheal intubation, respiratory rate, and oxygenation index in the ALI group were significantly higher than those in the non-ALI group (*p* < 0.05). It is noteworthy that, although the difference was not statistically significant, the proportion of non-survival patients in the ALI group (33.8%) was greater than that in the non-ALI group (19.4%). Moreover, Our testing revealed that the serum IL-34 level in the ALI group was significantly higher than that in the non-ALI group (*p* < 0.05) (Figure 4A).

**TABLE 4:** Baseline data of non-ALI group and ALI group.

Parameter	Non-ALI group (n = 36)	ALI group (n = 74)	*P* value
Age (years)	75 (59–81)	77 (71–82)	0.062
Gender (male/female)	20/16	43/31	0.800
Underlying diseases, n (%)			
Coronary heart disease	1 (2.8%)	12 (16.2%)	0.083
Hypertension	20 (55.6%)	47 (63.5%)	0.422
Type 2 diabetes	12 (33.3%)	13 (31.1%)	0.812
Chronic renal failure	2 (5.6%)	3 (4.1%)	1.000
Endotracheal intubation	14 (38.9%)	44 (59.5%)	**0.043**
Respiratory rate (per minute)	19 (16–24)	21 (18–24)	**0.045**
Heart rate (bpm)	96 (85–106)	98 (82–112)	0.804
Temperature (°C)	36.6 (36.5–37.2)	36.8 (36.5–37.6)	0.269
White blood count (10^9^/L)	12.3 (10.2–18.6)	13.4 (8.9–22.2)	0.909
Platelet count (10^9^/L)	173 (142–223)	190 (124–249)	0.719
Hematocrit (%)	33 ± 8	33.9 ± 8	0.107
Total bilirubin (μmmol/L)	16.8 (11.5–31.7)	19.5 (12.9–29.9)	0.369
Creatinine (μmmol/L)	94.8 (70.2–162.6)	107.3 (77–168.5)	0.649
GCS score	13 (9–15)	10 (6–15)	0.057
SOFA score	9 ± 3	10 ± 4	0.563
APACHE Ⅱ score	19 (14–24)	22 (17–28)	0.159
CRP (mg/L)	97.2 (43–178.1)	126.4 (56.9–175.3)	0.443
PaO_2_/FiO_2_ (mmHg)	349 (321–412)	222 (168–265)	**0.001**
The proportion of non-survival patients	19.4%	33.8%	0.120

The bolded *p*-value indicates a statistically significant difference (*p* < 0.05).

Subsequently, we further divided the ALI group into two subgroups based on the Berlin criteria: mild to moderate ALI and severe ALI. The baseline data are presented in Table 5. It is evident that the proportion of tracheal intubation and the oxygenation index are significantly higher in the severe ALI subgroup compared to the mild to moderate ALI subgroup. Notably, within the ALI group, serum IL-34 levels were significantly elevated in patients with severe ALI compared to those with mild to moderate ALI (*p* < 0.001) (Figure 4B).

**TABLE 5:** Baseline data of mild-to-moderate ALI and severe ALI subgroups.

Parameter	Mild to moderate ALI group (n = 62)	Severe ALI group (n = 12)	*P* value
Age (years)	77 (72–82)	75 (70–80)	0.528
Gender (male/female)	35/27	8/4	0.512
Underlying diseases, n (%)			
Coronary heart disease	10 (16.1%)	2 (16.7%)	1.000
Hypertension	41 (66.1%)	6 (50.0%)	0.462
Type 2 diabetes	19 (30.6%)	4 (33.3%)	1.000
Chronic renal failure	2 (3.2%)	1 (8.3%)	0.417
Endotracheal intubation	33 (53.2%)	11 (91.7%)	**0.031**
Mean arterial pressure (mmHg)	83.2 (69.8–96.7)	87.8 (79.2–102.1)	0.268
Respiratory rate (per minute)	21 (18–24)	23 (20–28)	0.155
Heart rate (bpm)	100 (63–112)	93 (79–111)	0.437
Temperature (°C)	37.0 (36.5–37.7)	36.8 (36.2–37.4)	0.398
White blood count (10^9^/L)	13.4 (9.1–22.1)	13.6 (5.1–23.5)	0.741
Platelet count (10^9^/L)	100 (121–234)	194 (127–270)	0.639
Hematocrit (%)	34.2 ± 8.0	32.7 ± 8.1	0.564
Total bilirubin (μmmol/L)	20.1 (12.8–33.0)	16.6 (14.1–25.6)	0.572
Creatinine (μmmol/L)	99.8 (76.6–168.5)	121.8 (84.5–214.7)	0.329
CRP (mg/L)	127.9 (56.9–175.3)	97.2 (54.6–182.2)	0.889
GCS score	10 (7–15)	10 (5–15)	0.935
SOFA score	10 ± 4	10 ± 4	0.842
APACHE Ⅱ score	22 (17–28)	23 (17–31)	0.752
PaO_2_/FiO_2_ (mmHg)	213 (168–254)	305 (125–553)	**0.031**

The bolded *p*-value indicates a statistically significant difference (*p* < 0.05).

Using 130.05 pg/ml as a cut-off value, ALI patients were categorized into high IL-34 and low IL-34 groups. Kaplan-Meier curves and log-rank tests were employed for comparison (Figure 4C). The analysis revealed that among ALI patients, those with elevated IL-34 levels had a 1.714-fold increased risk of reaching the endpoint of death compared to patients with lower IL-34 levels. Although the difference did not reach statistical significance (p = 0.173), this finding clinically suggests that elevated levels of IL-34 may be associated with a poorer prognosis in patients and should therefore be considered seriously in clinical practice. Furthermore, we conducted a univariate Cox regression analysis with IL-34 as the independent variable. The results indicated that the p-value for IL-34 exceeded 0.05. However, this does not imply that IL-34 lacks clinical relevance in these patients; rather, its impact may be obscured by various confounding factors. To strengthen our conclusion, we considered multiple potential confounders and applied multivariate Cox regression analysis. The results indicated that the hazard ratio of IL-34 remained close to 1 after adjusting for age, sex, and comorbidities, which further underscores the need for additional research into the prognostic value of IL-34.

## Discussion

The current study identifies IL-34 as an independent prognostic factor for survival in sepsis patients. A cutoff value of 130.05 pg/mL was established for stratifying patients at high risk of mortality. Additionally, IL-34 demonstrated a positive correlation with inflammatory biomarkers and disease severity. The prognostic value of IL-34 for survival was better than that of other inflammatory biomarkers including IL-6, CRP, and PCT. Our findings also suggest that IL-34 may serve as a valuable risk stratifier for sepsis-induced ALI. Additionally, we present the first preliminary investigation into the prognostic significance of IL-34 within this patient population.

Sepsis is widely recognized as a disease characterized by a systemic inflammatory response. Recent studies have increasingly highlighted IL-34 as a significant regulator of inflammatory responses and related diseases. Elevated levels of IL-34 have been associated with the severity of rheumatoid arthritis and poor prognosis (Zhou et al., 2016). Additionally, serum IL-34 levels in patients with systemic sclerosis have been reported to increase with the severity of interstitial lung disease (Kuzumi et al., 2018). Furthermore, IL-34 has been demonstrated to play a crucial role in hepatitis C-related liver fibrosis (Preisser et al., 2014). This study found that IL-34 is highly expressed in sepsis group and non-survival group, with expression levels correlating with disease severity. These findings align with previous research on IL-34 in inflammatory diseases. Additionally, Jacobs and Wiersinga (2018) identified IL-34 as an emerging factor in sepsis, suggesting its potential influence on pathogenic processes related to systemic inflammation and organ dysfunction. Research by Lin et al. (2018) also indicates that IL-34 is highly expressed in sepsis patients and may enhance survival rates and bacterial clearance in cases of polymicrobial sepsis, further underscoring the relationship between IL-34 and sepsis. However, a conflicting result observed by Lin et al. was that IL-34 levels did not correlate with APACHE II score or SOFA score in sepsis patients. This may be attributed to subtle differences in disease severity, ethnic background, sample size, and the criteria used for selecting sepsis patients. Another notable difference is that our study demonstrates a positive correlation between serum IL-34 levels and IL-6, PCT, and lactate levels during the onset of sepsis patients, which is also associated with prognosis. In contrast, Lin et al. did not investigate these relationships.

In the field of sepsis research, numerous biomarkers have been recognized, such as TNF-α, IL-8, IL-6, and CRP (Carcò et al., 2022; Li, 2024; Zeng et al., 2022). TNF-α, a classic pro-inflammatory factor, is rapidly released during the early stages of the inflammatory response and is significantly correlated with mortality in sepsis (Gharamti et al., 2022). IL-8, a potent chemokine, is closely associated with the risk of early mortality in sepsis and has a strong relationship with the SOFA score and multi-organ dysfunction (Livaditi et al., 2006). Our findings regarding IL-34 align with these observations: IL-34 is associated with sepsis severity and poor prognosis. Furthermore, we developed a univariate Cox regression model using age, gender, IL-34, IL-6, PCT, CRP, LAC, GCS score, SOFA score, and APACHE II score as independent variables. Our analysis indicated that the prognostic value of IL-34 surpasses that of IL-6 and CRP. It is important to clarify that our study specifically focused on IL-34 and did not aim to undermine the significance of established biomarkers. Rather, it sought to investigate the novel and underexplored role of IL-34 in sepsis. For future research, we recommend further examination of the use of IL-34 in conjunction with established biomarkers to enhance early diagnosis and risk stratification of sepsis.

Current research indicates that IL-34 is associated with the prognosis of various diseases. Previous studies have demonstrated that elevated levels of IL-34 correlate with poor outcomes in patients with heart failure, particularly those with chronic renal failure or insufficiency (Tao et al., 2017). Additionally, increased serum IL-34 levels upon admission are linked to a higher risk of acute ischemic stroke and are associated with diminished functional outcomes (Huang et al., 2021). Similarly, we explored the relationship between IL-34 and prognosis in sepsis patients. In our study, the ROC curve indicated that serum IL-34 exhibited enhanced clinical screening sensitivity and predictive value when compared to the SOFA score alone. Additionally, the results from the Kaplan-Meier survival analysis and the multifactorial Cox regression model indicated that IL-34 functions as an independent risk factor for 28-day mortality in sepsis patients, establishing a critical threshold of 130.05 pg/mL for identifying high-risk individuals. Therefore, these results suggest that serum IL-34 is associated with poor prognosis in sepsis patients.

ALI is a particularly dangerous complication of sepsis and significantly impacts patient prognosis. Notably, our study revealed that the proportion of ALI patients in the non-survival group was markedly higher than that in the survival group. Numerous previous studies have established a correlation between IL-34 and various respiratory diseases. For instance, Tang et al. (2024) reported a relationship between serum IL-34 levels and the severity in patients with community-acquired pneumonia. Additionally, another study indicated that IL-34 levels are elevated in patients with systemic sclerosis and are strongly linked to the incidence and severity of interstitial lung disease (Kuzumi et al., 2018). Furthermore, one investigation demonstrated that severe acute respiratory syndrome coronavirus 2 (SARS-CoV-2) infection significantly increases human IL-34 levels (Kaufmann et al., 2023). Consequently, we further categorized sepsis patients to explore the relationship between serum IL-34 and prognostic outcomes in those with ALI. In our study, we observed that the serum IL-34 level in the ALI group was significantly higher than that in the non-ALI group. Moreover, serum IL-34 levels were significantly elevated in patients with severe ALI compared to those with mild to moderate ALI. These findings suggest a potential association between IL-34 levels and the severity of ALI, which may provide valuable insights into the risk stratification of sepsis-induced ALI. Subsequently, we conducted a preliminary exploration of the prognostic value of IL-34 in this patient population using Kaplan-Meier curves and univariate Cox proportional hazards models. Although the results suggested that elevated IL-34 levels may be associated with poorer patient prognosis, none of the differences reached statistical significance. However, this does not imply that IL-34 lacks clinical relevance in these patients; rather, its effects may be obscured by various confounding factors. Therefore, we accounted for confounding variables such as age, sex, and comorbidities and applied multiple Cox regression analysis. The results indicated that, after adjusting for these confounding factors, the hazard ratio for IL-34 remained close to 1, underscoring the necessity for further studies on its prognostic value in sepsis-induced ALI. We recommend that future research consider additional confounding factors, such as pathogen type, nutritional status, and therapeutic interventions, and conduct larger prospective studies to further investigate the potential role of IL-34 in this pathological context.

This study has several limitations. First, the research was conducted at a single institution with a limited sample size, which may affect the generalizability and external applicability of the findings. Consequently, larger multicenter studies are warranted to validate these results and assess their clinical relevance. Second, the established IL-34 thresholds have not been externally validated or tested in independent cohorts or across multiple centers; thus, further studies are necessary to confirm their accuracy and applicability in diverse patient populations. Additionally, due to the limited availability of patient sera, we were unable to detect other biomarkers associated with sepsis, such as TNF-α, IL-1β, and IL-8. Furthermore, we did not evaluate the dynamic changes of IL-34 in all sepsis patients. Owing to various practical challenges, we only collected 22 consecutive serum samples from the original 115 patients. Although our results indicate that IL-34 levels changed during the first 5 days of hospitalization, we acknowledge that the small sample size may limit the statistical power and generalizability of our findings. It is essential to regard these results as an initial step in understanding IL-34 dynamics rather than a definitive conclusion. We sincerely hope that future studies with larger sample sizes will further validate and extend our observations.

In conclusion, IL-34 has been identified as an independent risk factor for predicting 28-day mortality in sepsis patients. It may serve as a potential biomarker for risk stratification in sepsis-induced ALI; however, the prognostic value of IL-34 in this particular patient population needs to be fully elucidated with a larger sample size and by considering more confounding factors in future studies.

## Data Availability

The raw data supporting the conclusions of this article will be made available by the authors, without undue reservation.
